# lncRNA AK054386 Functions as a ceRNA to Sequester miR-199 and Induce Sustained Endoplasmic Reticulum Stress in Hepatic Reperfusion Injury

**DOI:** 10.1155/2019/8189079

**Published:** 2019-11-13

**Authors:** Binghua Dai, Liang Qiao, Mingke Zhang, Lipeng Cheng, Ling Zhang, Li Geng, Changying Shi, Minfeng Zhang, Chengjun Sui, Weifeng Shen, Yanfu Sun, Qian Chen, Dong Hui, Yue Wang, Jiamei Yang

**Affiliations:** ^1^Department of Liver Transplantation, Eastern Hepatobiliary Surgery Hospital, Second Military Medical University, Shanghai, China; ^2^Department of Urology, Shanghai General Hospital, Shanghai Jiao Tong University, Shanghai, China; ^3^Department of Medical Genetics, Second Military Medical University, Shanghai, China; ^4^Department of Liver Surgery, Eastern Hepatobiliary Surgery Hospital, Second Military Medical University, Shanghai, China; ^5^Department of Histology and Embryology, Second Military Medical University, Shanghai, China; ^6^Department of Pathology, Eastern Hepatobiliary Surgery Hospital, Second Military Medical University, Shanghai, China; ^7^Shanghai Key Laboratory of Cell Engineering, Shanghai, China

## Abstract

Hepatic ischemia-reperfusion injury (IRI) is a very complex pathological process that is often associated with liver trauma and surgery, especially liver transplantation surgery. Although endoplasmic reticulum stress (ERS) plays a role in this process, the posttranscriptional regulators and the underlying mechanisms are still unclear. Here, we report that the lncRNA AK054386 was increased in hepatic IRI models. Furthermore, AK054386 can act as a “competing endogenous RNA (ceRNA)” and regulate ERS-related factors by binding and sequestering miR-199, which was shown to inhibit ERS in our previous report. Increased expression of AK054386, which might be mediated by activated NF-*κ*B, resulted in sustained ERS and increased cell apoptosis and death in hepatic IRI mouse and cellular models. In contrast, AK054386 inhibition had protective effects on these models. Our data indicate that AK054386 and miR-199 are critical players in hepatic IRI, and we broadened the scope regarding ceRNA mechanisms. We hope that our results will improve the understanding of hepatic IRI and may provide potential therapeutic targets.

## 1. Introduction

Hepatic ischemia-reperfusion injury (IRI) is a complex pathological process caused by temporarily interrupting the blood supply to the liver and is often associated with liver trauma, transplantation, and resection surgery. In particular, in liver transplantation surgery, the process of organ retrieval, cold preservation, and warm ischemia can result in severe liver injury and dysfunction. Therefore, IRI is one of the most challenging problems in this surgery [1–3]. This complex process results in the release of various inflammatory cytokines, such as interleukin-1*β* (IL-1*β*) and tumor necrosis factor-*α* (TNF-*α*), which can lead to hepatocyte death [4]. Although various factors such as pH, oxygen free radicals, ATP depletion, calcium influx, and activated inflammatory mediators [3] have been reported to play roles in IRI, the mechanisms of IRI are still obscure and may require the characterization of novel components and additional mechanisms.

Recent studies have found that endoplasmic reticulum stress (ERS) plays an important role in organ ischemia and reperfusion [5–7]. ERS can be caused by hypoxia and the lack of glucose, calcium overload, reactive oxygen species (ROS), and other factors. At the molecular level, the accumulation of misfolded proteins in the ER lumen causes the dissociation of GRP78 from three resident ER membrane proteins (inositol-requiring enzyme 1a (IRE1), protein kinase RNA-like endoplasmic reticulum kinase (PERK), and activating transcription factor 6 (ATF6)) and leads to their activation. Signal transduction cascades then regulate the transcription of ER stress-responsive genes, including GRP78 and proapoptotic transcription factor CHOP (C/EBP homologous protein), and inhibit new protein synthesis [6, 7]. Because hepatocytes contain a large amount of rough and smooth ER, disturbing ER homeostasis may contribute to hepatic IRI by mediating pathological changes. There is increasing evidence that ERS is involved in hepatic IRI [7, 8]. Additionally, more and more reports have pinpointed to noncoding RNAs as important regulators of gene expression [9–13]. However, the roles and underlying mechanisms of related noncoding RNAs in ERS-mediated hepatic IRI are still not clear. Our previous report demonstrated that one of the most abundant miRNAs in hepatocytes, miR-199a-5p, was elevated in both bile acid- and thapsigargin- (TG-) stimulated cultured hepatocytes as well as in the livers of bile duct-ligated mice [14]. The elevated miR-199a-5p levels disrupted sustained ERS and prevented hepatocytes from undergoing bile acid- or TG-induced cell death by directly targeting and suppressing GRP78, IRE1, and ATF6 [14]. Because ERS plays an important role in hepatic IRI, we speculated whether miR-199-5p is associated with hepatic IRI. In the current work, we investigated the role of miR-199-5p in hepatic IRI and tried to elucidate the underneath mechanism associated with long noncoding RNA (lncRNA). We hope that our results may improve the understanding of hepatic IRI and may provide experimental reference for better diagnosis and treatment of this disease in the future.

## 2. Materials and Methods

### 2.1. Mice and Cell Lines

C57BL/6 mice (females at 9–11 weeks) were purchased from the SMMU Laboratory Animal Center and used in accordance with the institutional guidelines for animal care. All mouse experiments were performed according to the Guide for the Care and Use of Laboratory Animals issued by the Ministry of Science and Technology of the People's Republic of China. All mouse experiments were approved by the committee of Experimental Animal Administration of the Second Military Medical University (SMMU).

The mouse hepatocyte line BNL-CL2 was obtained from the American Type Culture Collection (ATCC) (Manassas, VA, USA) and maintained using the provided guidelines. Please refer to the supplementary information for detailed methods and materials.

### 2.2. Induction of the Hepatic IRI Models in Mice and Cells

Hepatic IRI was induced following a common protocol that was described previously [15, 16]. To generate a mouse hepatic IRI model, a microvascular clamp was applied to the Glisson system. After 1 h of hepatic ischemia, the clamp was removed for the designated period of reperfusion for further analysis. The hepatic ischemia models underwent only 1 h of ischemia without reperfusion. Sham-operated controls underwent the same procedure except for vascular occlusion. For all the mouse experiments, 5 mice were in each group (sham-operated controls, ischemia, and IRI).

Cells were cultured for 8 h in ischemia-mimic medium (10.0 mmol/L KCl, 98.5 mmol NaCl, 0.9 mmol/L NaH_2_PO_4_, 20.0 mmol/L HEPES, 6.0 mmol/L NaHCO_3_, 1.8 mmol/L CaCl_2_, 1.2 mmol/L MgSO_4_, and 40.0 mmol/L sodium lactate pH 6.8) and in a gas mixture of 1.0% O_2_, 5.0% CO_2_, and 94% N_2_ to mimic ischemia. To simulate reperfusion, the cells were cultured under normal conditions at 5% CO_2_ and 37°C for 3 h and the ischemia-mimic medium was then replaced with normal culture medium. See the detailed methods in the supplementary information.

### 2.3. Serum Measurements

Mouse serum levels of aspartate aminotransferase (AST) and alanine aminotransferase (ALT) were measured using a commercial AST Activity Assay Kit (BioVision) and ALT Activity Assay Kit (BioVision) according to the manufacturer's instructions at the SMMU Animal Experiment Center.

### 2.4. Gene Overexpression and Knockdown

Lentiviral vectors, plasmid vectors, miRNA mimics, and siRNAs were utilized for gene overexpression and knockdown. Please refer to the supplementary information for detailed methods and materials. For the *in vitro* gene overexpression and knockdown experiments in the IRI cell model, molecular exchange or phenotypes were tested 48 h after transfection unless otherwise specified.

### 2.5. Apoptosis Assays

Apoptosis was analyzed by flow cytometry analysis and TUNEL [8]. See the detailed methods in the supplementary information.

### 2.6. Cytotoxicity Assays

Hepatocyte damage was determined by analyzing the level of LDH in cell culture supernatants using semiautomated and routine clinical methods.

### 2.7. RNA Immunoprecipitation Assay

The AK054386-MS2 and AK054386-Mut-MS2 plasmids were cloned, and the MS2bp-MS2bs-based RNA immunoprecipitation (RIP) assay was performed using an EZ-Magna RIP Kit (Merck Millipore Headquarters, Billerica, MA, USA) according to the manufacturer's instructions and previous reports [17] with modifications.

### 2.8. Chromatin Immunoprecipitation Assay

ChIP assays were performed using the EZ-Magna ChIP A/G Kit (Millipore) according to the manufacturer's instructions. Chromatin was immunoprecipitated using a control anti-IgG antibody (Santa Cruz Biotechnology, USA) or a p105/p50 antibody (Cell Signaling Technology, USA). And the ChIP-derived DNA samples were quantified by quantitative real-time PCR (qRT-PCR). The sequence of the AK054386 promoter region was acquired from the UCSC website. Detailed methods and primer sequences used for PCR are provided in the supplementary information.

Detailed methods for qRT-PCR, the luciferase reporter assay, and Western blot (WB) analysis are also provided in the supplementary information.

### 2.9. Statistical Analysis

Most data are presented as the mean ± S.D. of at least 3 independent experiments. ANOVA analysis and Fisher's exact test or two-tailed Student's *t*-test were performed for statistical comparisons between experimental groups, and *P* < 0.05 was considered statistically significant.

## 3. Results

### 3.1. miR-199 Levels Are Decreased but ERS-Related Factors Are Elevated in Hepatic Ischemia and Reperfusion

**Figure 1:**
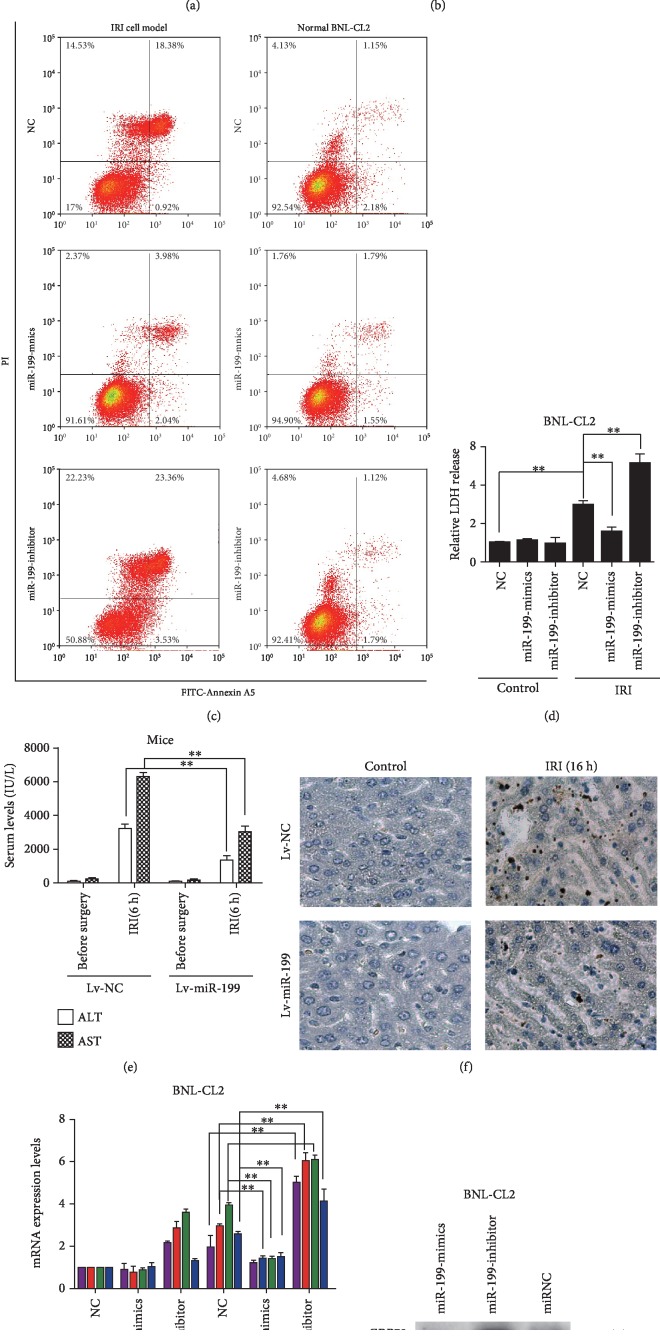
miR-199 protects hepatocytes from IRI *in vitro* and *in vivo*. The relative levels of miR-199a-5p in mouse liver tissues from the control, ischemia, and IRI groups. RNA levels were quantified by qRT-PCR and normalized to U6. Five mice were analyzed in each group. ANOVA, ^∗∗^*P* < 0.01. (b) The relative levels of ER stress-related genes as measured by qRT-PCR and normalized to GAPDH. ANOVA, ^∗∗^*P* < 0.01. (c) miR-199 impacts BNL-CL2 apoptosis in an IRI cell model. Apoptosis rates were assayed by flow cytometry. (d) BNL-CL2 cell death levels were assayed by LDH release. (e) Mouse serum ALT and AST levels were analyzed and presented as the mean ± S.D. (*n* = 5). ANOVA, ^∗∗^*P* < 0.01. (f) Representative light photomicrographs of TUNEL-stained sections of mouse liver tissue from control mice and mouse hepatic IRI models. Five mice were analyzed in each group. (g, h) Expression of ER stress-related genes, as measured by (g) qRT-PCR and (h) Western blot in the mouse BNL-CL2 cell line. The qRT-PCR results were normalized to GAPDH. Con and BNL-CL2 cells were cultured under normal conditions, and IRI and BNL-CL2 cells were cultured under IRI conditions. Data are shown as the mean ± S.D. of 5 independent experiments. ANOVA, ^∗^*P* < 0.05 and ^∗∗^*P* < 0.01.

To investigate the role of miR-199 in hepatic IRI, we first constructed an *in vivo* hepatic IRI mouse model by temporarily blocking the mouse Glisson system using a previously described protocol [15]. Histological analysis showed hepatocyte necrosis in the hepatic IRI mouse model compared to the sham-operated controls and mice treated with only hepatic ischemia without reperfusion ([Supplementary-material supplementary-material-1]). Next, we examined miR-199a-5p expression levels by qRT-PCR and found a statistically significant decrease (*P* < 0.01) in expression in the hepatic IRI mouse model compared to that in the sham-operated controls and hepatic ischemia mice, although there was no obvious change between control and ischemic mice (Figure 1(a)). We found that ERS genes targeted by miR-199a-5p were increased in the hepatic IRI mice (Figure 1(b)). We also successfully created a hepatic IRI cell model in the mouse hepatocyte line BNL-CL2 by incubating cells in ischemia-mimic media and low O_2_ conditions. We found that apoptosis was increased in these hepatic IRI cells ([Supplementary-material supplementary-material-1]) and that miR-199a-5p expression was decreased, while the expression of the ERS genes was increased in the hepatic IRI cell model ([Supplementary-material supplementary-material-1] and [Supplementary-material supplementary-material-1]). All these data indicate that miR-199 may play a role in hepatic IRI.

### 3.2. miR-199 Protects Hepatocytes from IRI *In Vitro* and *In Vivo*

To study the function of miR-199 in hepatic IRI, we manipulated the levels of miR-199 in BNL-CL2 cells. As shown, miR-199 overexpression robustly suppressed apoptosis in the BNL-CL2 IRI cell model and cells with decreased miR-199 expression showed increased apoptosis (Figure 1(c)). The LDH release experiment also showed that miR-199 overexpression decreased the cell death rate, while miR-199 inhibition promoted cell death (Figure 1(d)). To further examine the role of miR-199 in hepatic IRI *in vivo*, we overexpressed miR-199 in mice by lentiviral injection, followed by surgical hepatic IRI 72 h later. Overexpression of miR-199 had a protective role in the hepatic IRI mouse model by decreasing serum AST and ALT levels (Figure 1(e)) and suppressing hepatocyte apoptosis (Figure 1(f)). Furthermore, the expression levels of the ERS-related genes GRP78, ATF6, IRE1a, and CHOP were increased after miR-199 inhibition and decreased after miR-199 overexpression at both the RNA and protein levels (Figures 1(g) and 1(h)). These data indicate that miR-199 may play a role in hepatic IRI by inhibiting ERS-related genes.

### 3.3. The Decrease in miR-199 Is Mediated by the Increase in AK054386 in Hepatic IRI

**Figure 2:**
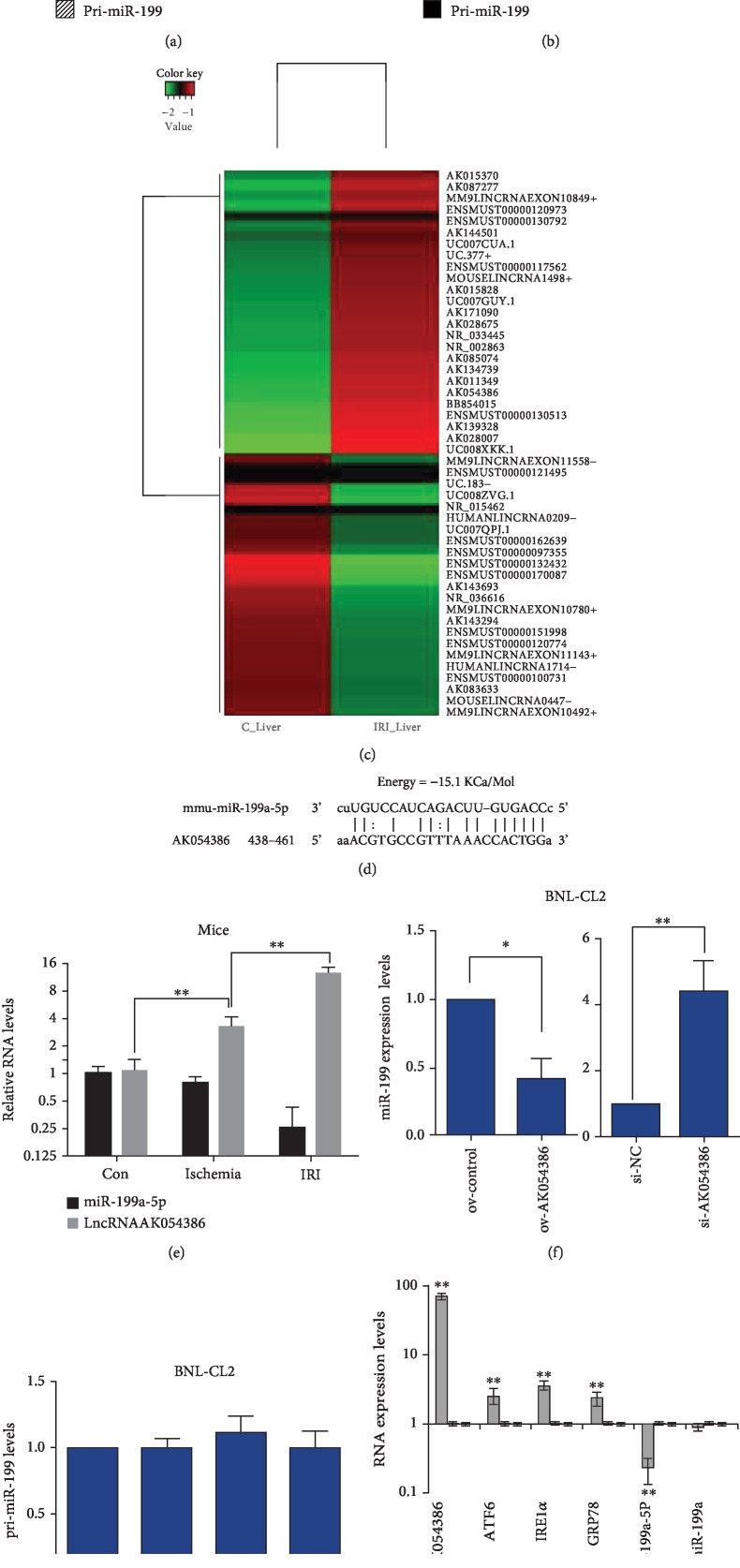
The decrease in miR-199 is mediated by increased AK054386 in hepatic IRI. (a, b) The expression levels of the mature and premature forms of miR-199 were tested by qRT-PCR in both the (a) cell and (b) mouse hepatic IRI models. The values were normalized to U6. Student's *t*-test, ^∗^*P* < 0.05. (c) Hierarchical clustering analysis of microarray data mined from the GEO database (GSE47412). (d) Putative binding sites for miR-199a-5p on AK054386 predicted with Miranda software. (e) The relative levels of AK054386 and miR-199 in mouse liver tissue from the control, ischemia, and IRI groups. RNA levels were quantified by qRT-PCR and normalized to GAPDH or U6. Five mice were analyzed in each group. ANOVA, ^∗∗^*P* < 0.01. (f, g) Relative levels of the (f) mature and (g) premature forms of miR-199 in BNL-CL2 cells transfected with the AK054386 overexpression plasmid or knockdown siRNAs tested using qRT-PCR analysis. Data are shown as the mean ± S.D. of 6 independent experiments. ANOVA, ^∗^*P* < 0.05 and ^∗∗^*P* < 0.01. (h) The expression levels of related factors in BNL-CL2 cells as measured by qRT-PCR analysis. GAPDH or U6 was used as the endogenous control. Data are shown as the mean ± S.D. of three independent experiments. Student's *t*-test, ^∗^*P* < 0.05 and ^∗∗^*P* < 0.01.

To investigate whether the decrease in miR-199 in hepatic IRI is regulated at a transcriptional or posttranscriptional level, we examined the premature form of miR-199 and found interesting results; although the expression of mature miR-199 decreased robustly, there were no significant changes in pri-miR-199 in either the cellular or mouse hepatic IRI models (Figures 2(a) and 2(b)). This result demonstrates that the decrease in miR-199 in hepatic IRI does not occur at the transcriptional level but rather at the posttranscriptional level. These results suggested that miR-199 may be regulated by a common RNA posttranscriptional regulation model: competing endogenous RNA (ceRNA) mechanism, which means that lncRNAs can bind and sequester certain miRNAs, acting as a sponge; therefore, lncRNAs compete with miRNAs' other targets, ultimately reducing the suppression of these targets [18–20]. Using bioinformatic methods, we mined published data [21] and found that some lncRNAs were greatly increased in hepatic IRI (Figure 2(c)). AK054386 was among the most increased lncRNAs in hepatic IRI and had putative miRNA response elements (MREs) or binding sites for miR-199a-5p (Figure 2(d)), as identified by RNA22 program analysis (https://cm.jefferson.edu/rna22/Interactive/). More importantly, we found that AK054386 expression levels were increased in hepatic IRI mouse models (Figure 2(e)). Moreover, AK054386 overexpression decreased the levels of mature miR-199 but had no impact on its premature form, pri-miR-199. In contrast, knocking down AK054386 increased the expression of mature miR-199 but not its premature form (Figures 2(f) and 2(g), [Supplementary-material supplementary-material-1] E-F). Furthermore, overexpression of AK054386 affected ERS-related factors (Figure 2(h)). All these results indicate that AK054386 mediates miR-199 expression and may play an important role in hepatic IRI.

### 3.4. AK054386 Functions as a ceRNA and Interacts with miR-199

**Figure 3:**
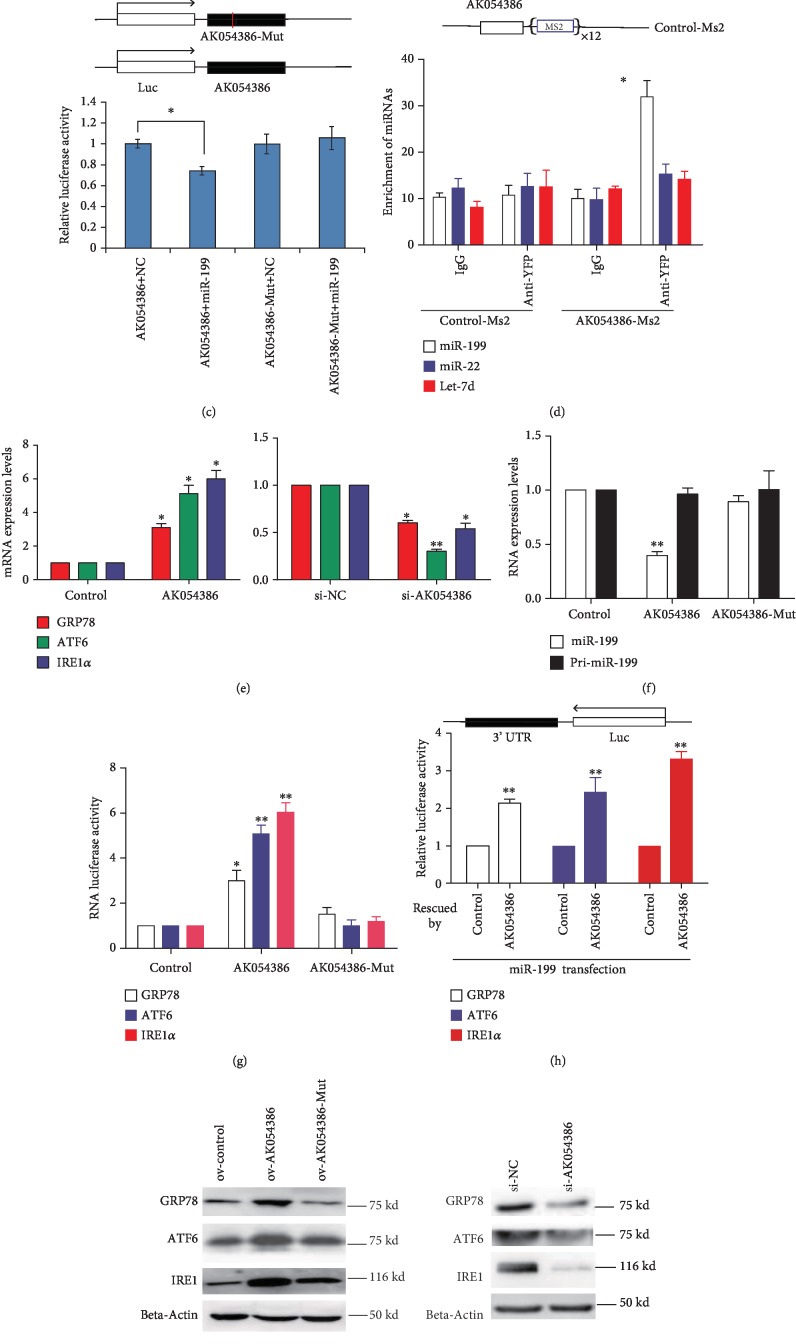
AK054386 functions as a ceRNA and interacts with miR-199. Subcellular localization of AK054386 by qRT-PCR of the subcellular fractionations of BNL-CL2 cells. The *in situ* expression of AK054386 was analyzed by FISH using a FITC-AK054386 RNA probe or a FITC-scrambled sequence RNA probe (green), and the nuclear DNA was counterstained with DAPI (blue). (b) The relative levels of AK054386 were analyzed by qRT-PCR after miR-199 overexpression or inhibition. Data are shown as the mean ± S.D. of 6 independent experiments. ANOVA, ^∗∗^*P* < 0.01. (c) Target validation using luciferase reporters in HEK293 cells. Mut: mutant; NC: scrambled RNA; Luc: luciferase. ANOVA, ^∗^*P* < 0.05. (d) RIP experimental results in BNL-CL2 cells using the MS2-MS2-BP system. The enrichment of miRNAs was measured by qRT-PCR. (e) Changes in the previously reported targets of miR-199 after AK054386 overexpression or knockdown as analyzed by qRT-PCR. Data are shown as the mean ± S.D. of 6 independent experiments. ANOVA, ^∗∗^*P* < 0.01 and ^∗^*P* < 0.05. (f, g) The levels of miR-199 and its target genes after transfection of wild-type AK054386 or mut-AK054386 expression vectors. The RNA levels were tested by qRT-PCR. Data are shown as the mean ± S.D. of 6 independent experiments. ANOVA, ^∗∗^*P* < 0.01 and ^∗^*P* < 0.05. (h) AK054386 overexpression can directly compete with GRP78, ATF6, and IRE1a for miR-199 binding. Student's *t*-test, ^∗∗^*P* < 0.01. (i) GRP78, ATF6, and IRE1a protein levels were affected by the changes in wild-type AK054386 as measured by Western blot. (j) ChIP results show the binding of NF-*κ*B to the AK054386 promoter. For all qRT-PCR analyses in this figure, GAPDH or U6 was used as an endogenous control. Data are shown as the mean ± S.D. The results are statistics from three independent experiments unless otherwise indicated. Student's *t*-test, ^∗∗^*P* < 0.01. All the experiments were done in BNL-CL2 cells except for the luciferase reporter assays in (h), which were done in 293 cells. (k) Model for the AK054386-related regulatory loop in the modulation of hepatocyte ERS in hepatic I/R. In hepatic IRI, overactivated UPR causes sustained ERS, which induces the activation of nuclear factor-*κ*B (NF-*κ*B1). NF-*κ*B can bind to the AK054386 promoter and induce its transcription. This lncRNA then sequesters miR-199, resulting in the upregulation of GRP78, ATF6, and IRE1a, which promotes aggravated and sustained ER stress. This positive feedback response causes hepatocyte apoptosis and cell death.

To validate the hypothesis that miR-199 may be regulated by AK054386 via a ceRNA mechanism, we first investigated the subcellular localization of AK054386. We found that AK054386 is mainly distributed in the cytoplasm (Figure 3(a)) using subcellular fractionation and fluorescence *in situ* hybridization (FISH) methods. As a ceRNA that interacts with miR-199, AK054386 should also be a target of miR-199. In addition, we found that AK054386 indeed is a target of miR-199 because miR-199 overexpression decreased AK054386 expression levels, while inhibition of miR-199 upregulated AK054386 expression (Figure 3(b)). We then constructed a luciferase-AK054386 reporter vector and cotransfected it with miR-199 mimics into a HEK293 cell model. The miR-199 mimic significantly reduced the luciferase activities of the AK054386 reporters compared with the NC (Figure 3(c)). To validate the direct binding of AK054386 and miR-199, we first constructed an AK054386-MS2 fusion RNA expression vector. We then cotransfected the AK054386-MS2 vector with a vector expressing MS2-BP, a protein that specifically binds to the MS2 sequence [17, 22], and we finally performed RIP assays. miR-199 was enriched in the AK054386-MS2 group after RIP compared to that in the control-MS2 group (Figure 3(d)), suggesting that AK054386 can bind to miR-199 thus pulling miR-199 down. Furthermore, AK054386 overexpression increased the expression levels of the ERS-related factors GRP78, ATF6, and IRE1a, as well as other targets of miR-199, while knockdown of AK054386 decreased these factors at both the RNA (Figure 3(e)) and protein (Figure 3(i)) levels. Specifically, the ability of AK054386 to regulate miR199 (Figure 3(f)) and ERS-related miR-199 target genes (Figure 3(g)) is dependent on the miR-199 binding sites in the lncRNA, as mutation of these sites in AK054386 had weaker effects on the downstream factors than that in wild-type AK054386. Finally, we performed direct competition experiments by overexpressing miR-199 withGRP78, ATF6, and IRE1a luciferase reporters, followed by AK054386 overexpression and determined whether AK054386 could compete with the luciferase reporter constructs and increase the luciferase activity. The results showed that AK054386 functions as a ceRNA of these mRNAs by competing for miR-199 binding (Figure 3(h)). All these results indicate that AK054386 functions as a ceRNA and interacts with miR-199 in hepatic IRI.

### 3.5. Increased AK054386 in Hepatic IRI Is Mediated by NF-*κ*B

We next studied the mechanism underlying the upregulation of AK054386 in hepatic IRI. Nuclear factor-*κ*B (NF-*κ*B) is an important factor downstream of the ERS pathway and is a central player in ischemia-reperfusion-mediated injury [23–25]. Interestingly, using the JASPAR (http://jaspar.genereg.net) bioinformatic analysis tool, two putative NF-*κ*B binding sites were found 800 bp upstream of the AK054386 TSS ([Supplementary-material supplementary-material-1] A-C). In addition, ChIP analysis showed that NF-*κ*B p50 subunits were enriched in the AK054386 promoter compared to those in the control group with the IgG antibody (Figure 3(j)). These data suggest that NF-*κ*B contributes to increased AK054386 expression during hepatic ischemia and reperfusion. The increase in AK054386 expression then sequesters miR-199, resulting in the upregulation of GRP78, ATF6, and IRE1a, which then induces ERS and causes hepatocyte injury (Figure 3(k) and [Supplementary-material supplementary-material-1]).

### 3.6. AK054386 Affects Hepatic Injury from Ischemia and Reperfusion *In Vivo* and *In Vitro*

**Figure 4:**
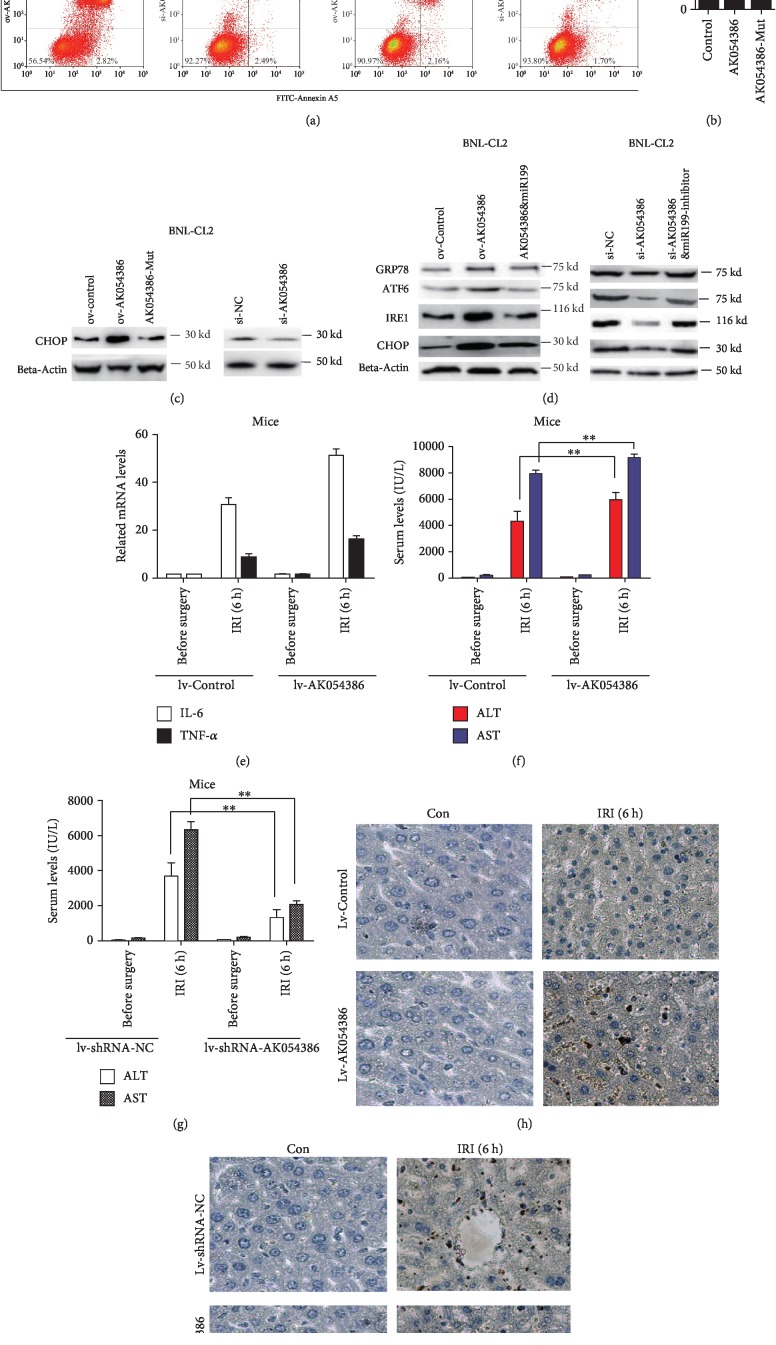
AK054386 affects hepatic injury from ischemia and reperfusion *in vivo* and *in vitro*. (a) AK054386 impacts apoptosis in the BNL-CL2 IRI cell model. Apoptosis rates were assayed by flow cytometry. Representative data from three independent experiments are shown. (b) BNL-CL2 cell death levels were assayed by LDH release. Data are shown as the mean ± S.D. of three independent experiments. (c, d) The protein levels of ER-related genes were analyzed by Western blot. (e) The relative RNA expression levels of inflammatory cytokines (IL-6 and TNF-*α*) of liver tissues from mouse hepatic IRI models after lentivirus infection. 5 mice were analyzed in each group. The RNA levels were measured by qRT-PCR and normalized to GAPDH. Data are shown as the mean ± S.D. of three independent experiments. ANOVA, ^∗^*P* < 0.05 and ^∗∗^*P* < 0.01. (f, g) Mouse serum ALT and AST levels were analyzed and presented as the mean ± S.D. Five mice were analyzed in each group. ANOVA, ^∗∗^*P* < 0.01 and ^∗^*P* < 0.05. (h, i) Representative light photomicrographs of TUNEL-stained sections of mouse liver tissue from control mice and mouse hepatic IRI models after lentivirus infection. Five mice were analyzed in each group.

The function of AK054386 in hepatic IRI also needs to be investigated. We found that overexpression of AK054386 increased the apoptosis and cell death rate in the BNL-CL2 IRI cell model, while knockdown of AK054386 decreased the apoptosis and cell death rate (Figure 4(a)). Interestingly, the impact of AK054386 on hepatocyte cell death was dependent on its miR-199 binding sites, as mutation of these sites had no effect on LDH release (Figure 4(b)). Furthermore, AK054386 affected the expression of the ERS-related factor CHOP; this effect was also dependent on the miR-199-binding sites in AK054386 (Figure 4(c)). More interestingly, AK054386 overexpression increased CHOP, GRP78, ATF6, and IRE1a expression and these effects were rescued by miR-199 overexpression. In contrast, AK054386 knockdown downregulated the levels of these proteins, which were rescued by miR-199 inhibition (Figure 4(d)). For the *in vivo* experiments, control lentivirus, AK054386-overexpressing lentivirus, or lentivirus expressing AK054386 shRNAs were intravenously injected 72 h before surgical induction of hepatic IRI. In addition, we found that after AK054386 overexpression, the hepatic IRI mouse model showed more necrosis and edema degeneration ([Supplementary-material supplementary-material-1]), higher mRNA levels of the inflammatory cytokines IL-6 and TNF-*α* in the liver (Figure 4(e)), and higher ALT and AST levels in the serum (Figure 4(f)). Additionally, higher levels of hepatocyte apoptosis were also observed after AK054386 overexpression (Figure 4(h)) compared to those in mice injected with the control lentivirus. In contrast, injection of a lentivirus expressing AK054386 shRNAs showed protective effects on the hepatic IRI mouse model, as the serum ALT and AST levels were decreased and cell apoptosis in the livers of these mice was suppressed (Figures 4(g) and 4(i)). All these data indicate that AK054386 plays an important role in hepatic IRI via miR-199 by mediating the ERS pathway; these results were confirmed in both mouse and cell models.

## 4. Discussion

In this study, we found a new factor, lncRNA AK054386, that plays a critical role in hepatic ischemia and reperfusion injury (IRI) by functioning as a ceRNA to sponge miR-199 in both hepatic IRI *in vivo* and *in vitro* mouse models. AK054386 was highly increased while miR-199 was decreased under hepatic IRI conditions. In contrast, a decrease in AK054386 expression or miR-199 overexpression showed protective effects on hepatic cells through ERS-related pathways. We also found that the increase in AK054386 may be caused by the activation of nuclear factor-*κ*B in hepatic IRI because NF-*κ*B1 can bind to the AK054386 promoter. Our results explored the molecular mechanism of hepatic IRI to some extent and may provide new strategies to solve this problem in the future.

In recent years, there are more and more reported evidences indicating that miRNA can induce mRNA degradation and/or mRNA translation suppression through imperfect matching to the 3′UTR of target mRNAs [9–11]. And miRNA-mediated posttranscriptional regulation networks play critical roles in liver diseases [26, 27]. As one of the abundant miRNAs in normal hepatocytes [28–30], miR-199 can disrupt sustained ERS by inhibiting GRP78, IRE1, and ATF6 and preventing hepatocytes from undergoing bile acid- or TG-induced cell death [14]. However, the role of miR-199 in hepatic IRI is still not fully known. In this study, we found that miR-199 expression levels decreased during hepatic IRI but not in the ischemia-only group. This may be due to the different mechanisms of ischemic injury and ischemia-reperfusion injury. We also found that miR-199 protected hepatocytes from IRI. Although more phenotyping studies using clinical samples are needed, our results indicate that miR-199 is an important factor in hepatocytes and that its regulation is critical for liver health.

In our current study, we also identified that lncRNA AK054386 is a regulator of miR-199 and plays a critical role in hepatic ischemia-reperfusion injury (IRI). Recently, some reports have shown that the lncRNAs MALAT1 and UCA1 can participate in cardiac IRI [31, 32]. However, the roles of lncRNAs in hepatic IRI are still not fully understood and only a few reports have studied changes in total lncRNA levels by expression profiling [21]. Investigating specific lncRNAs and elucidating the mechanisms will help better understand the role of lncRNAs in IRI. In this paper, we demonstrated that the expression of AK054386 was elevated in hepatic cells during IRI and that manipulating AK054386 expression affected hepatic IRI both *in vivo* and *in vitro*. We also found that NF-*κ*B1 could bind to the AK054386 promoter, thus partially elucidating the mechanism by which AK05438 expression is increased in hepatic IRI. Moreover, we found that AK054386 functions as a ceRNA to bind and sequester miR-199 and plays a critical role in hepatic ischemia-reperfusion injury (IRI).

The competing endogenous RNA (ceRNA) mechanism is a recently reported RNA-RNA interaction model which is theoretically common in many bioprocesses [18–20]. Indeed, ceRNA mechanisms have been reported to be involved in development, cancer, and plenty of other situations [33–35]. However, whether this mechanism was related to hepatic IRI was unknown until now. Here, we reported that AK054386 can function as a ceRNA to bind miR-199 and increase the expression levels of GRP78, IRE1, and ATF6, which are key factors in the ERS pathway. Thus, AK054386 can promote ERS through a ceRNA mechanism in hepatic IRI. Our data also indicate that lncRNA and miRNAs may regulate the cascade of signaling events which may drive the progression of hepatic IRI through. Knowing that RNA-RNA interactions are universal in cells, more systematic approaches are needed to obtain more meaningful AK054386 downstream target genes and to obtain other lncRNAs in addition to AK054386 that functions as ceRNAs in hepatic IRI, for example, the combination of high-throughput sequencing results of all mRNA, lncRNA, and microRNAs at specific stages of hepatocytes in the IRI environment. Overall, our results may shed light on the molecular mechanism of hepatic IRI and may provide new targets for IRI treatment.

## 5. Conclusions

In this study, we found that a new factor, the lncRNA AK054386, plays a critical role in hepatic ischemia-reperfusion injury (IRI) by functioning as a ceRNA to sponge miR-199 in both *in vivo* and *in vitro* hepatic IRI mouse models. AK054386 was highly increased while miR-199 was decreased under hepatic IRI conditions. In contrast, a decrease in AK054386 expression or miR-199 overexpression showed protective effects on hepatic cells through ERS-related pathways. We also found that increased AK054386 expression may be caused by activation of nuclear factor-*κ*B in hepatic IRI because we found that NF-*κ*B1 can bind to the promoter of AK054386.

## Data Availability

We declare that the materials described in the manuscript, including all relevant raw data, will be freely available to any scientist for use in noncommercial applications, without breaching participant confidentiality.

## Abbreviations

IRI: Hepatic ischemia-reperfusion injury
ERS: Endoplasmic reticulum stress
SMMU: Second Military Medical University
AST: Aspartate aminotransferase
ALT: Alanine aminotransferase.

## Supplementary Materials

Supplementary Materials Figure S1: expression levels of miR-199 and ERS-related factors in hepatic IRI. Figure S2: increased AK054386 expression in hepatic IRI is mediated by NF-*κ*B. Figure S3: AK054386 increases liver tissue necrosis and inflammation.
