# Supplementary material for: lncRNA AK054386 Functions as a ceRNA to Sequester miR-199 and Induce Sustained Endoplasmic Reticulum Stress in Hepatic Reperfusion Injury
Source: Oxid Med Cell Longev. 2019 Nov 13;2019:8189079. doi: 10.1155/2019/8189079 (PMC6885273; doi:10.1155/2019/8189079)
Supplement: Supplementary Materials — Figure S1: expression levels of miR-199 and ERS-related factors in hepatic IRI. Figure S2: increased AK054386 expression in hepatic IRI is mediated by NF-κB. Figure S3: AK054386 increases liver tissue necrosis and inflammation. [file 8189079.f1.pdf]

Supplementary Information

lncRNA AK054386 functions as a ceRNA to sequester miR-199 and induce sustained endoplasmic reticulum stress in hepatic reperfusion injury

Authors

Binghua Dai[a,1], Liang Qiao[a,1], Mingke Zhang[e,1], Lipeng Cheng[a], Ling Zhang[d], Li Geng[a], Changying Shi[a], Minfeng Zhang[a], Chengjun Sui[a], Weifeng Shen[a], Yanfu Sun[b], Qian Chen[c], Dong Hui[f], Yue Wang[c,]*, Jiamei Yang[a,]*

Affiliations

a. Department of Liver Transplantation, Eastern Hepatobiliary Surgery Hospital, Second Military Medical University, Shanghai, China
b. Department of Liver Surgery, Eastern Hepatobiliary Surgery Hospital, Second Military Medical University, Shanghai, China
c. Research Center of Developmental Biology, Second Military Medical University, Shanghai, China
d. Department of Medical Genetics, Second Military Medical University, Shanghai, China
e. Department of Urology, Shanghai General Hospital, Shanghai Jiaotong University, Shanghai, China
f. Department of Pathology, Eastern Hepatobiliary Surgery Hospital, Second Military Medical University, Shanghai, China

This section contains:

Supplementary Figures 1and 2

Supplementary Methods

1 These authors contribute equally to this work.

* Corresponding authors

Supplementary Figure 1:

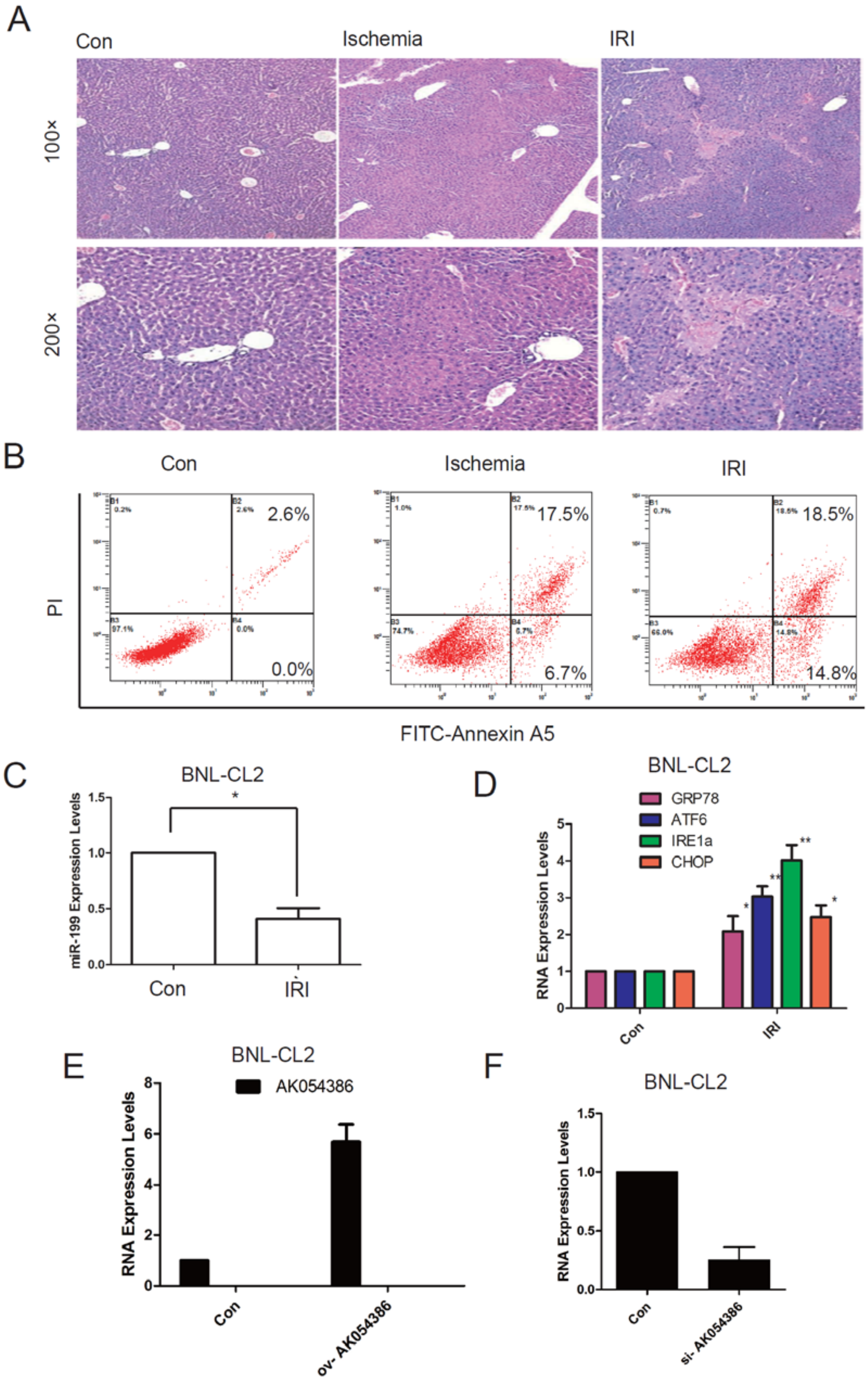

Figure S1. Expression levels of miR-199 and ERS-related factors in hepatic IRI.

(A). Representative hematoxylin-and-eosin (HE) stained sections of liver tissues from the sham-operated controls (Control), the mice suffering from hepatic ischemia without reperfusion (Ischemia) and the hepatic IRI mouse model (IRI). More serious necrosis was observed in the IRI group compared with that in the other two groups. **(B)** Apoptosis rates were assayed by flow cytometry and indicated the successful construction of the hepatic IRI cell model in the mouse BNL-CL2 cell line. Data are representative of three independent experiments. **(C and D)** The relative expression levels of miR-199a-5p and ER stress-related genes measured by qRT-PCR and normalized to GAPDH in the hepatic IRI cell model using the mouse BNL-CL2 cell line. **(E-F)** The relative expression levels of AK054386 after its overexpression(**E**) or knock-down(**F**), the RNA levels were measured by qRT-PCR and normalized to GAPDH in BNL-CL2 cell line. For all the qRT-PCR analysis in this figure, data are shown as the means±S.D. of three independent experiments. * $P<0.05$, ** $P<0.01$.

Supplementary Figure 2:

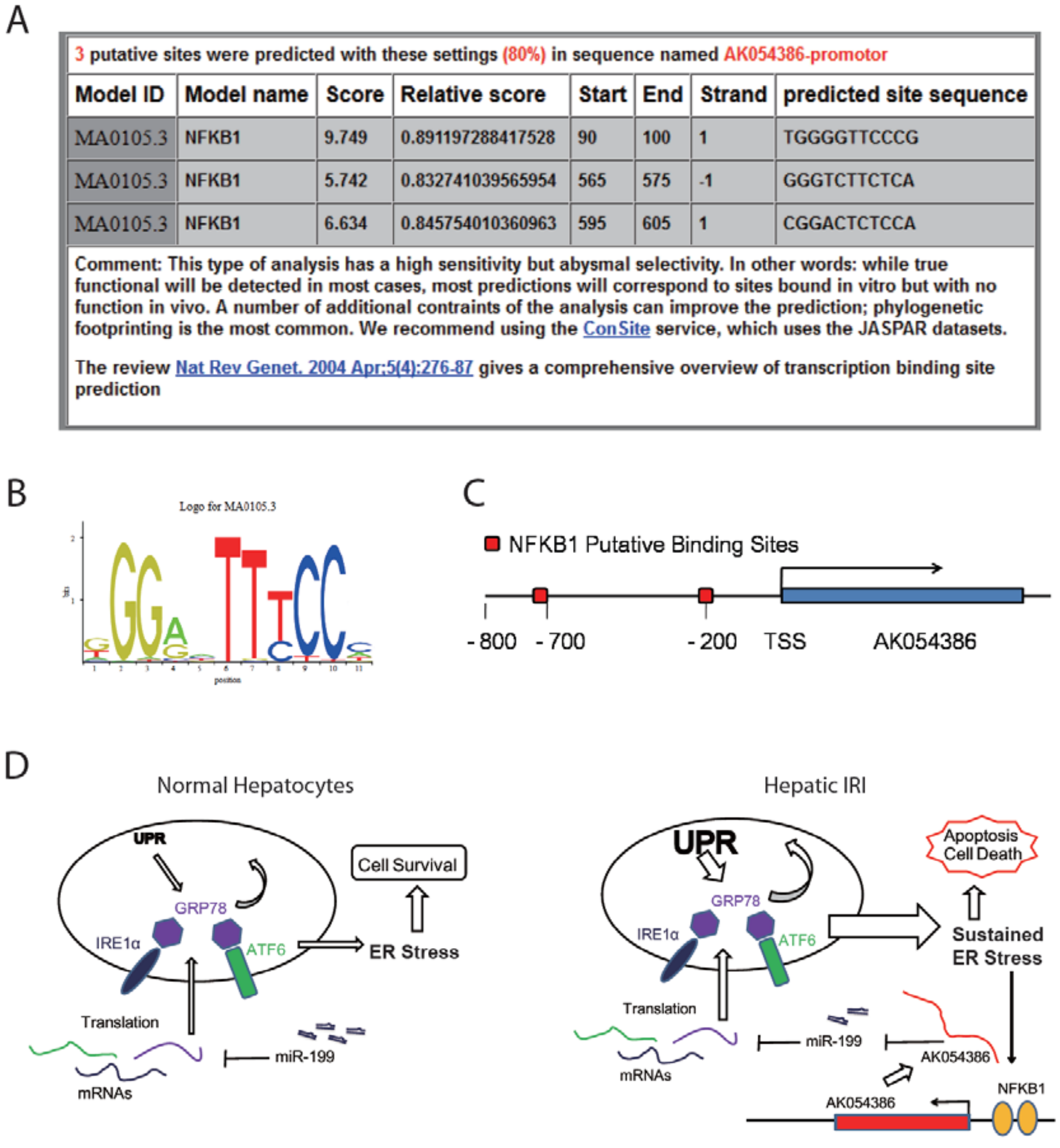

3 putative sites were predicted with these settings (80%) in sequence named AK054386-promotor

Model ID	Model name	Score	Relative score	Start	End	Strand	predicted site sequence
MA0105.3	NFKB1	9.749	0.891197288417528	90	100	1	TGGGGTTCCCG
MA0105.3	NFKB1	5.742	0.832741039565954	565	575	-1	GGGTCTTCTCA
MA0105.3	NFKB1	6.634	0.845754010360963	595	605	1	CGGACTCTCCA

Comment: This type of analysis has a high sensitivity but abysmal selectivity. In other words: while true functional will be detected in most cases, most predictions will correspond to sites bound in vitro but with no function in vivo. A number of additional contraints of the analysis can improve the prediction; phylogenetic footprinting is the most common. We recommend using the ConSite service, which uses the JASPAR datasets.

The review Nat Rev Genet. 2004 Apr;5(4):276-87 gives a comprehensive overview of transcription binding site prediction

Figure S 2. Increased AK054386 expression in hepatic IRI is mediated by NF-κB. (A) Putative NF-κB binding sites in the AK054386 promoter predicted by the JASPAR website. **(B)** NF-κB binding site sequences were provided by the JASPAR website. **(C)** Putative NF-κB binding sites in the AK054386 promoter. **(D)** AK054386 functions as a ceRNA to sequester miR-199 and induce sustained ER stress in hepatic IRI. The unfolded protein-induced ER stress response could result in the elevation of ER mediator mRNAs. In normal hepatocytes, miR-199 could negatively regulate ER stress by targeting the mRNAs of ER-related factors, which

promotes hepatocyte survival. In hepatic IRI, over-activated UPR causes sustained ERS, which induces the activation of Nuclear Factor-κB (NFKB1). NF-κB can bind to the AK054386 promoter and induce its transcription. This LncRNA then sequesters miR-199, resulting in the up-regulation of GRP78, ATF6 and IRE1a, which promotes aggravated and sustained ER stress. This positive feedback response causes hepatocyte apoptosis and cell death.

Supplementary Figure 3:

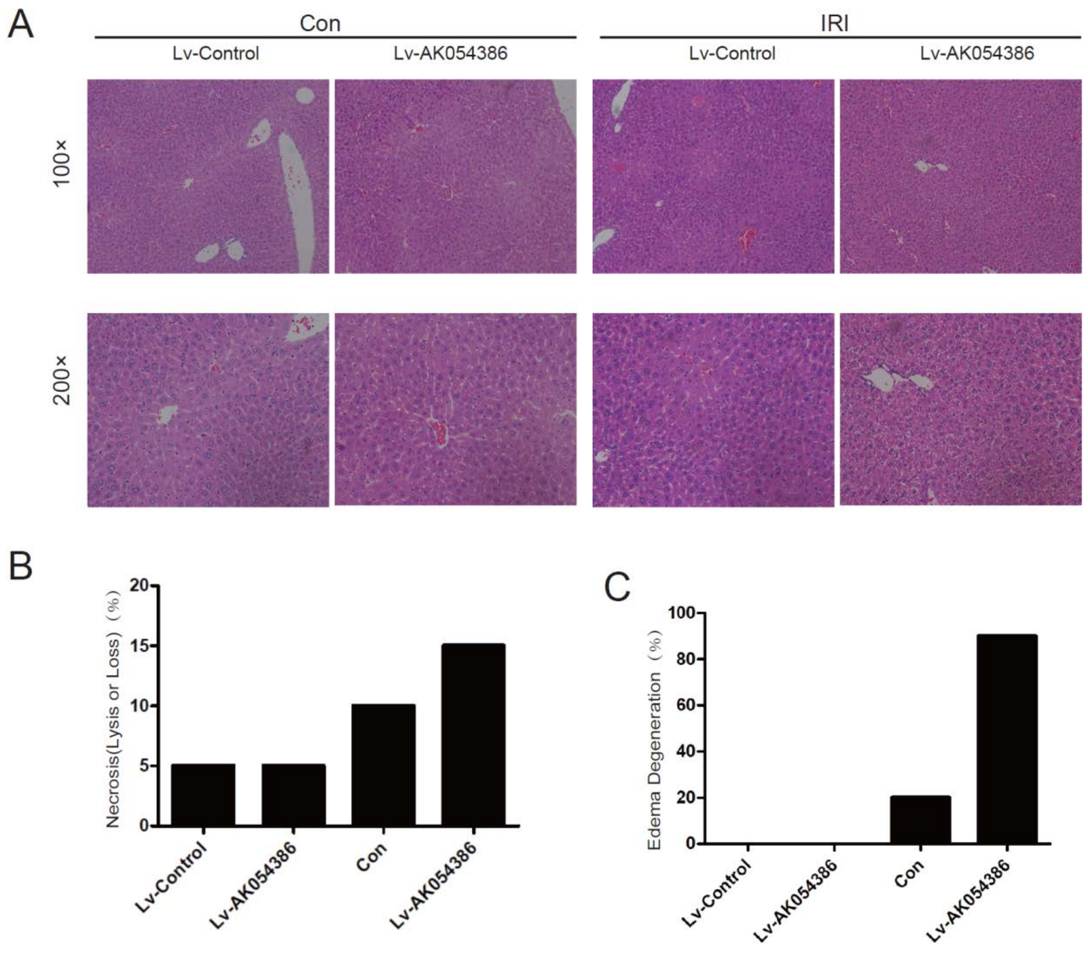

Figure S3. AK054386 increase liver tissue necrosis and inflammation.

(A) Representative hematoxylin-and-eosin (HE) stained sections of liver tissues from control mice and mouse hepatic IRI models after lentivirus infection. 5 mice were analyzed in each group. **(B) Quantitative analysis of necrosis(lysis or loss) of the HE stained**

sections of liver tissues from A. (C) Quantitative analysis of edema degeneration（%）of the HE stained sections of liver tissues from A.

Supplementary Methods

Mice, cell lines and reagents. C57BL/6 mice (Female at 9–11 weeks) were purchased from the SMMU Laboratory Animal Center and were used in accordance with the Institutional guidelines for animal care. The cell lines were cultured following a common protocol that was described in our previous manuscript (Dai BH, et al., 2013). The mouse hepatocyte line BNL-CL2 was obtained from the American Type Culture Collection (ATCC, Manassas, VA, USA) and was maintained in minimum essential medium (MEM) supplemented with 10% fetal bovine serum (FBS, Invitrogen, Carlsbad, CA, USA), 100 mM MEM nonessential amino acids, 2 mM l-glutamine ,1 mM sodium pyruvate , and 50 U/ml penicillin & streptomycin (all from Invitrogen). The human embryonic kidney epithelial cell line HEK293 (ATCC) was maintained in DMEM medium containing 10% FBS. SiRNA and miRNA inhibitors/mimics were purchased from Genepharma (Shanghai, China). Anti-ATF6, anti-GRP78 (Bip), anti-IRE1A, anti-CHOP and anti-NFKB1 antibodies were purchased from Cell Signaling Technology (CST, Beverly, MA, USA). Unless specifically indicated, all chemicals were purchased from Sigma Chemical Co. (St. Louis, MO, USA).

Induction of hepatic IRI model in mice

Hepatic IRI was induced following a common protocol that is described previously (Abe et al., 2009, Kim J.Y. et al., 2017). Briefly, mice underwent a midline laparotomy after anesthetization with chloral hydrate, and the hepatic hilum was dissected. A microvascular clamp was applied to the Glisson system for constructing

the hepatic IRI model. The clamp was removed and the abdominal wall was closed after 1 h of hepatic ischemia. After a designated period (1 h/3 h/6 h) of reperfusion, the whole blood or liver samples of the mice were collected following anesthesia for further study (histological analysis, TUNEL assay, and other molecular analyses). The hepatic ischemia models underwent only 1 h of ischemia without reperfusion. Sham-operated controls underwent the same procedure except for vascular occlusion. For all the mouse experiments, 5 mice were in each group (Sham-operated controls, ischemia and IRI).

Induction of hepatic IRI model in cells.

A three gas incubator was set with the following gas concentrations: (1) 1.0% O_2, (2) 5.0% CO_2 and (3) 94% N_2. The next day, the normal medium on the cultured mouse hepatocyte line BNL-CL2 was changed to ischemia-mimic medium(10.0 mmol/L KCl, 98.5 mmol NaCl, 0.9 mmol/L NaH_2PO_4, 20.0 mmol/L HEPES, 6.0 mmol/L $NaHCO_3$, 1.8 mmol/L $CaCl_2$, 1.2 mmol/L $MgSO_4$, and 40.0 mmol/L sodium lactate pH 6.8). Then, the cells were placed in to the three gas incubator and cultured for 8 h for ischemia simulation. To simulate reperfusion, the cells were cultured under normal conditions at 5% CO_2 and 37°C for 3 h and the ischemia-mimic medium was then replaced with normal culture medium.

Serum measurements. Mice serum levels of Aspartate aminotransferase (AST) and alanine aminotransferase (ALT) were measured using a commercial AST Activity Assay Kit (Biovision) and ALT Activity Assay Kit (Biovision) according to the manufacturer’s instructions at the SMMU Animal experiment center.

Interleukin 6 (IL-6) and C-Reactive Protein (CRP) measurements.

The liver tissues were homogenized with ice-cold PBS. ELISA kits were applied for measurement of IL-6 and CRP levels in liver tissue homogenates in accordance with

instructions (Xitang Biotechnology, Shanghai, China).

Subcellular fractionation. Cytosolic fractions and nuclear fractions of BNL-CL2 cell were isolated and collected with a PARIS kit AM1921 (AmTX) according to the manufacturer's instructions to investigate the subcellular distribution of AK054386. Then, the total RNA was extracted from the cytosolic fractions and nuclear fractions respectively, followed by cDNA synthesis and qRT-PCR .

Gene over-express and knock-down. Lentiviral vectors were purchased from Genepharma (Shanghai, China). For miR-199 overexpression, the lentivirus contained the premiR-199 sequences, whereas for AK054386 overexpression, the whole sequence was included in the lentivirus. The lentiviral infection experiments were performed according to the manufacturer's instructions and was described in our previous manuscript (Dai BH, et al., 2013). For the *in vivo* experiments, the lentivirus was injected intravenously 3 days before hepatic IRI modeling surgery. The plasmid of AK054386 and AK054386-Mut were purchased from ObioTechnology (Shanghai, China). The siRNAs were synthesized by Genepharma (Shanghai, China) with the following sequence: 5'- GUGGCUGGUUAAUAUCUCATT -3'. The miRNA mimics were synthesized by Genepharma (Shanghai, China). The plasmid and miRNA mimics and siRNA were transfected into cells using Lipofectamine™ 3000 Reagent（Thermo Fisher Scientific，USA ）following the instrument.

The sequence of wild-type AK054386 (miR-199 binding site is highlighted)：

1 acaaaaaatg acaagagaga tgaaaacgtt tgattatttt ctcagtgtat ttttgtaaaa

61 aatatataaa gggggtgtta atcggtgtaa atcgctgttt ggatttcctg attttataat

121 agggtggctg gttaatatct cacacagttt gaaaaatcag cccctggttt ctccatgttt

181 acacttcaat ctgcaggctt cttaaagtga cagtatccct taacctgcca ccggtttcca

241 cctttcaacc ccctggtctt ataaggggag gagagttcag cccagcacca taatgcttta

301 aaaaaaaaaa ttttttttt ttaacgaatt gctgttctgt ccagaggttt ttaaaactgg

361 tgcattcaca gcaaaaaggg attctgtagc tttaacttgt aaaccacatc tttttgcac

421 tttttttat aaagcaaaaa cgtgccgttt aaaccactgg atctatctaa atgccgattt
481 gagttcgcga cacaatgtac tgcgttttca ttcttgtatt tgactattta atcctttcta
541 cttgtcgcta aatataattg tttcagtcct atggcatggt gatagcatat gtgttcaggt
601 ttatagctgt tgtgtttaaa gattgaaaaa agtggaaaac atctttgtac atttaagtct
661 gtattataat aagcaaaaag attgtgtgta tgtatgttta atataacatg acaggcacga
721 ggacgcctgc cttttaagag gcagttccgt taagggtttt tgttttaaaa ctcttttttt
781 tcctttcttt ttttatttct tttttttaac catccatcct gtgcaatatg ccgtgtagaa
841 tatttgtctt aaaattcaag gccacaaaaa aaagcaaaaa aaaaaaaaca aaaaaaaaaa
901 caaaaaacaa tatttggggg aag

The sequence of AK054386-Mut（mutated sequence is highlighted）:

1 acaaaaaatg acaagagaga tgaaaacgtt tgattatttt ctcagtgtat ttttgtaaaa
61 aatatataaa gggggtgtta atcggtgtaa atcgctgttt ggatttcctg attttataat
121 agggtggctg gttaatatct cacacagttt gaaaaatcag cccctggttt ctccatgttt
181 acacttcaat ctgcaggctt cttaaagtga cagtatccct taacctgcca ccggtttcca
241 cctttcaacc ccctggtctt ataaggggag gagagttcag cccagcacca taatgcttta
301 aaaaaaaaaa ttttttttt ttaacgaatt gctgttctgt ccagaggttt ttaaaactgg
361 tgcattcaca gcaaaaaggg attctgtagc tttaacttgt aaaccacatc ttttttgcac
421 tttttttat aaagcaaa tcgatgccgtag aaactaggct ctatctaa atgccgattt
481 gagttcgcga cacaatgtac tgcgttttca ttcttgtatt tgactattta atcctttcta
541 cttgtcgcta aatataattg tttcagtcct atggcatggt gatagcatat gtgttcaggt
601 ttatagctgt tgtgtttaaa gattgaaaaa agtggaaaac atctttgtac atttaagtct
661 gtattataat aagcaaaaag attgtgtgta tgtatgttta atataacatg acaggcacga
721 ggacgcctgc cttttaagag gcagttccgt taagggtttt tgttttaaaa ctcttttttt
781 tcctttcttt ttttatttct tttttttaac catccatcct gtgcaatatg ccgtgtagaa
841 tatttgtctt aaaattcaag gccacaaaaa aaagcaaaaa aaaaaaaaca aaaaaaaaaa
901 caaaaaacaa tatttggggg aag

Apoptosis assays. Apoptosis was analyzed by flow cytometry analysis following a common protocol that was described in our previous manuscript (Dai BH, et al., 2013). Cells from each sample were tested using Annexin V-PE apoptosis detection kit (BioVision, Inc., USA) by a BD FACSCalibur flow cytometer (BD Bioscience, San Jose, CA, USA) according to the manufacturer's instructions. Triplicate samples （30000 events/ sample）were acquired and analyzed using the FACSDiva software (version 4.1.2; BD Biosciences). Apoptosis was also analyzed by TUNEL which evaluated DNA fragmentation as reported in a previous protocol (Mosbah et al., 2012; Zaouali et al., 2013).

Cytotoxicity assays. Hepatocyte damage was determined by analyzing the LDH in cell culture supernatants using semi-automated and routine clinical methods.

RNA preparation, reverse transcription and quantitative real-time-PCR (qRT-PCR). Methods in this part are also following a common protocol that was described in our previous manuscript(Dai BH, et al., 2013).. Briefly, total RNA was extracted using TRIzol reagent (Invitrogen). Taqman probes and primer sets (AB, Foster City, CA, USA) were used for testing the miRNA levelsaccording to the manufacturer's instructions. For mRNA analysis, the Reverse Transcription System Kit (Promega, Madison, WI, USA) was used for the first-strand cDNA generatation . ThePower SYBR Green PCR Master Mix (Applied Biosystems)was used for real-time PCR testing on a StepOne Plussystem (Applied Biosystems). GAPDH mRNA levels or U6 snoRNA were used as internal normalization controls.

The following primer pairs used for PCR:

GAPDH, 5'-CGGATTTGGTCGTATTGGG-3' and

5'-CTG GAAGATGGTGATGGGATT-3';

U6, 5'-GCTTCGGCAGCACATATACTAAAAT-3' and

5’- CGCTTCACGAATTTGCGTGTCAT -3’

LncR-AK054386 , 5’-AAGGGGGTGTTAATCGGTGT-3’and

5’- AACATGGAGAAACCAGGGGC-3’

GRP78, 5’-GGAGGAGGACAAGAAGGA-3’ and

5’-AGTGAAGGCGA CATAGG A-3’;

IRE1A, 5’-GGTCTGAGGAAGGTGATG-3’ and

5’-CATAGAGGCTGGTAGAGTATT-3’;

CHOP, 5’-CCTCACTCTCCAGATTCCA-3’ and

5’-AGCCGTTCATTCTCTTCAG-3’;

ATF6, 5’-AACAAGACCACAAGACCAA-3’ and

5’ -AGGAGGAACTGACGAACT-3’;

pri-miR-199 A1, 5’-CCGCTCTGTCCCTTCTGACG-3’ and

5’-AAACCCTGCCTCCTGCTCC-3’;

premiR-199A1, 5’-GCCAACCCAGTGTTCAGACTAC-3’

5’-GCCTAACCAATGTGCAGACTACT-3’;

pri-miR-199A2, 5’-TGCCCAGTCTAACCAATGTGC-3’

and 5’-AGCTGAATGCAACCCCTGG-3’; and

premiR-199A2, 5’-AGCTTCTGGAGATCCTGCTCC-3’ and

5’-TGCTCTCCCTTGCCCAGTCT-3’;

miR-199a-5p, 5’-TTATTACCCAGGCAGAC-3’ and

5’- ACCGAGTGCGAACTGTGGCGAT-3’

RIP Assay.

The AK054386-MS2 and AK054386-Mut-MS2 plasmid were cloned using following primers: 5’- CTATAGGGAGACCCAATCGGTGTAAATCGCTGTTTG-3’ and 5’- GCTCGGTACCAAGCTCATATTGCACAGGATGGATGGT -3’. Using the EZ-Magna RIP Kit (Millipore), the MS2bp-MS2bs-based RIP assay was performed

according to previous reports(Gong and Maquat, 2011) with modifications.

Luciferase reporter assay. Methods in this part are also following a common protocol that was described in our previous manuscript (Dai BH, et al., 2013) with modifications. Briefly, wild-type AK054386 and MIR199 putative binding site mutated AK054386 (AK054386-Mut) were clone into pMIR-REPORT™ Luciferase using following primers: 5'- AGGTTTAAACAGTTA TCGGTGTAAATCGCTGTTTG and 5'- GATCCTTTATTAAGCTCATATTGCACAGGATGGATGGT-3'.

These reporters were transfected into HEK293 and BNL-CL2 cells in 96 well plates using Lipofectamine 3000 (Invitrogen). The cells were then co-transfected with wild-type (WT) or mutant(Mut) reporter vector（80 ng）,and pRL-TK-Renilla-luciferase plasmid（8 ng）which is the internal control and the indicated RNAs (final concentration of 50 nM) were co-transfected into the cells. The luciferase activities were measured 48h after tansfection using the Dual-Luciferase Reporter Assay System (Promega). As reported previously, the data were normalized by dividing the firefly luciferase activity by the Renilla luciferase activity.

Chromatin immunoprecipitation assay. Methods in this part are also following a common protocol that was described in our previous manuscript (Dai BH, et al., 2013) with modifications. EZ-Magna ChIP A/G Kit (Merck Millipore Headquarters, Billerica, MA, USA) was used for ChIP assays according to the manufacturer's instructions. Chromatin was immunoprecipitated using p105/p50 antibody (Cell Signaling Technology , USA) or an anti-IgG antibody as the control (Santa Cruz Technologies, Santa Cruz, CA, USA). ChIP-derived DNA was quantified using qRT-PCR (Applied Biosystems). AK054386's promoter region was acquired from the UCSC website. The following primer sequences were used for ChIP quantification:

(1) primer pair 1, sense 5'-TATAGGTGTAATTGAGAAGACCC-3' and anti-sense 5'-AAAAAGAAGATAAAGAAACGAGG-3'; (2) primer pair 2, sense

5’-TAGTGTAGGATAATATAAAAAGC-3’ and anti-sense,
5’-GAGGGGGTAAAAATAAACGGGTC-3’; and (3) control primer, sense
5’-GAGGTCTCGTATTTGCTGCATCGTA-3’ and anti-sense
5’-GCTAATTTCCTTCTCCACCCCAACCA-3’.

Fold enrichments were calculated from the apparent IP efficiency (ratio of ChIP enriched DNA over control IgG input DNA) and normalized to the levels at a control region.

Western blotting analysis. Identical quantities of cell protein lysates were prepared in a 1x sodium dodecyl sulfate buffer and were separated by sodium dodecyl sulfate polyacrylamide gel electrophoresis. The proteins were then transferred onto polyvinylidene fluoride membranes following fist and second antibody incubation.

Statistical analysis. Most Data are presented as the mean±S.D. of at least 3 independent experiments. ANOVA analysis and Fisher’s exact test or two-tailed Student’s t-test were performed for statistical comparisons between experimental groups, and $P < 0.05$ was considered statistically significant.

References

Abe, Y., Hines, I.N., Zibari, G., Pavlick, K., Gray, L., Kitagawa, Y., and Grisham, M.B. (2009). Mouse model of liver ischemia and reperfusion injury: method for studying reactive oxygen and nitrogen metabolites in vivo. Free Radic Biol Med *46*, 1-7.

Dai BH, Geng L, Wang Y, Sui CJ, Xie F, Shen RX, Shen WF, Yang JM.(2013) microRNA-199a-5p protects hepatocytes from bile acid-induced sustained endoplasmic reticulum stress. Cell Death & Disease. 4. e604.

Gong, C., and Maquat, L.E. (2011). lncRNAs transactivate STAU1-mediated mRNA decay by

duplexing with 3' UTRs via Alu elements. Nature *470*, 284-288.

Kim, J. Y. , Lee, D. Y. , Kang, S. , Miao, W. , Kim, H. , & Lee, Y. , et al. (2017). Bilirubin nanoparticle preconditioning protects against hepatic ischemia-reperfusion injury. Biomaterials, 133, 1-10.

Mosbah, I.B., Zaouali, M.A., Martel, C., Bjaoui, M., Abdennebi, H.B., Hotter, G., Brenner, C., and Rosello-Catafau, J. (2012). IGL-1 solution reduces endoplasmic reticulum stress and apoptosis in rat liver transplantation. Cell Death Dis *3*, e279.

Zaouali, M.A., Boncompagni, E., Reiter, R.J., Bejaoui, M., Freitas, I., Pantazi, E., Folch-Puy, E., Abdennebi, H.B., Garcia-Gil, F.A., and Rosello-Catafau, J. (2013). AMPK involvement in endoplasmic reticulum stress and autophagy modulation after fatty liver graft preservation: a role for melatonin and trimetazidine cocktail. J Pineal Res *55*, 65-78.
